# Role of 11β-hydroxysteroid dehydrogenase type 1 in the development of atopic dermatitis

**DOI:** 10.1038/s41598-020-77281-x

**Published:** 2020-11-19

**Authors:** Noo Ri Lee, Beom Jun Kim, Chung Hyeok Lee, Young Bin Lee, Solam Lee, Hyun Jee Hwang, Eunjung Kim, Sung Hee Kim, Min-Geol Lee, Sang Eun Lee, Gareth G. Lavery, Eung Ho Choi

**Affiliations:** 1 Department of Dermatology, Yonsei University Wonju College of Medicine, Wonju, Republic of Korea; 2 Department of Dermatology, Severance Hospital, Cutaneous Biology Research Institute, Yonsei University College of Medicine, Seoul, Republic of Korea; 3 Department of Dermatology, Cutaneous Biology Research Institute, Yonsei University College of Medicine, Gangnam Severance Hospital, Seoul, Republic of Korea; 4 Institute of Metabolism and Systems Research, College of Medical and Dental Sciences, University of Birmingham, Birmingham, B15 2TT UK

**Keywords:** Chronic inflammation, Skin diseases

## Abstract

Glucocorticoids (GCs) are potent anti-inflammatory drugs, the secretion of which is mediated and controlled by the hypothalamic–pituitary–adrenal axis. However, they are also secreted de novo by peripheral tissues for local use. Several tissues express 11β-hydroxysteroid dehydrogenase 1 (11β-HSD1), including the skin. The inactive GC cortisone is converted by 11β-HSD1 to active GC cortisol, which is responsible for delayed wound healing during a systemic excess of GC. However, the role of 11β-HSD1 in inflammation is unclear. We assessed whether 11β-HSD1 affects the development of atopic dermatitis (AD) in vitro and in vivo. The expression of 11β-HSD1 in the epidermis of AD lesions was higher than that in the epidermis of healthy controls. Knockdown of 11β-HSD1 in human epidermal keratinocytes increased the production of thymic stromal lymphopoietin. In an oxazolone-induced mouse model of AD, localized inhibition of 11β-HSD1 aggravated the development of AD and increased serum cytokine levels associated with AD. Mice with whole-body knockout (KO) of 11β-HSD1 developed significantly worse AD upon induction by oxazolone. We propose that 11β-HSD1 is a major factor affecting AD pathophysiology via suppression of atopic inflammation due to the modulation of active GC in the skin.

## Introduction

Cortisol is an endogenous glucocorticoid (GC) in humans, whose release is stimulated by the activation of the hypothalamic–pituitary–adrenal (HPA) axis by various stressors^1^. Exogenous GCs have been used as a potent anti-inflammatory drug for chronic inflammatory diseases, including atopic dermatitis (AD)^2,3^. The main anti-inflammatory mechanism of GCs is the suppression of inflammatory gene transcription factors such as nuclear factor-kappa B (NF-κB) and activator protein-1^4^.

Apart from systemic GC production elicited by the HPA axis, extra-adrenal GCs are synthesized de novo in various tissues, including the brain, colon, heart, lung, thymus, and skin^5–9^. Epidermal and follicular keratinocytes, melanocytes, and dermal fibroblasts in the skin have glucocorticosteroidogenic activity^5,6,10–13^.

11β-Hydroxysteroid dehydrogenases (11β-HSDs) regulate the peripheral availability of GCs. There are two isoforms of 11β-HSD: 11β-HSD1 converts inactive cortisone into cortisol^14,15^, whereas 11β-HSD2 inactivates cortisol to cortisone^16,17^. Epidermal keratinocytes, dermal fibroblasts, sebocytes, and sweat gland cells in the skin, and the outer root sheath cells of hair follicles express 11β-HSD1^7,18–20^, and this expression is increased by ultraviolet (UV) B and UVC but not UVA irradiation^17,21^. Increased 11β-HSD1 expression leads to increased availability of local GCs and increased suppression of inflammatory responses in keratinocytes^22^. The expression of 11β-HSD1 mRNA was reportedly higher in human dermal fibroblasts (HDFs) obtained from a donor-matched photo-exposed outer upper arm than in HDFs obtained from the photo-protected inner upper arm^19^. The expression and activity of 11β-HSD1 were elevated in biopsies of photo-exposed skin (outer lower arm) compared to those in biopsies of donor-matched photo-protected skin (inner upper arm) in vivo^23^. Inflammation in response to hapten-induced dermatitis was exacerbated and the response to topical 11-dehydrocorticosterone was attenuated in keratinocyte-specific *11β-HSD1* (*HSD11b1*) knockout (KO) mice compared with the reactions in WT mice. These findings confirmed that GCs exert their immunosuppressive effect through 11β-HSD1^24^. Therefore, 11β-HSD1 is not only involved in the cutaneous side effects caused by excess GCs but also plays a key role in local anti-inflammatory activity in keratinocytes.

AD is one of the most common inflammatory diseases of the skin. It is characterized by severe pruritus and its multifactorial pathogenesis involves a complex combination of epidermal barrier defects, T helper cell type 2 (Th2)-skewed inflammation, and neuroendocrinal dysregulation^14,25–28^. The involvement of GCs in inflammatory skin diseases such as AD has been proposed recently. Several studies have described that patients with AD show a blunted response to psychological stress with lower cortisol production compared with normal controls^29–31^. In mice, the absence of GC receptors in the epidermis results in skin barrier defects and increased susceptibility to cutaneous inflammation^32^. The role of 11β-HSD1 in AD has not been studied yet. However, evidence from recent studies supports the notions that 11β-HSD1 is involved in inflammatory cytokine expression in keratinocytes and that it plays an important role in AD exacerbation by modulating local GC availability^14^. The expression of thymic stromal lymphopoietin (TSLP) is significantly increased in the epidermis of keratinocyte-specific *Hsd11b1* KO compared to that in WT mice^33^. Moreover, GCs suppress the upregulation of TSLP in normal human epidermal keratinocytes (NHEK) transfected with 11β-HSD1 small interfering RNA (siRNA). Thus, endogenous GCs activated by the homeostatic activation of 11β-HSD1 might regulate TSLP production^33^.

In this study, we aimed to determine the impact of regulation of endogenous GCs by 11β-HSD1 on the development of AD in vitro and in vivo*.*

## Results

### Knockdown of 11β-HSD1 by siRNA transfection increases TSLP production in NHEKs

**Figure 1:**
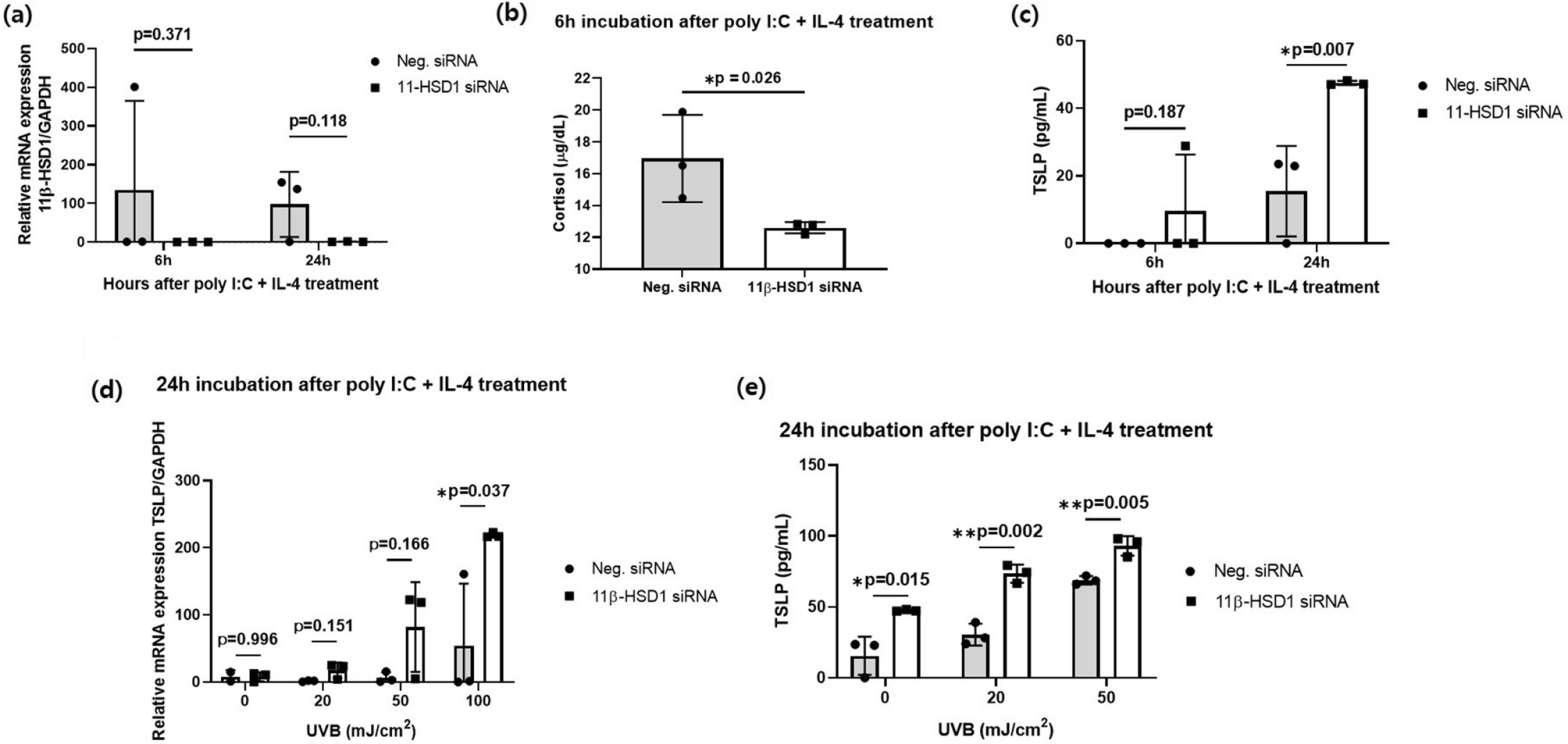
Effect of 11β-HSD1 knockdown on TSLP production in NHEK cells. (**a**) We transfected NHEK with either 11β-HSD1 siRNA or negative siRNA then mRNA expression of 11β-HSD1 was quantified by qPCR after six and 24 h incubations with poly I:C and IL-4. (**b**) Cortisol in culture medium measured after six-hour incubation with poly I:C and IL-4. (**c**) Amount of TSLP in culture media measured after six and 24 h incubations with poly I:C and IL-4. (**d**) mRNA expression of TSLP quantified by qPCR after irradiation with 0, 20, 50, 100 mJ/cm^2^ UVB. (e) Amount of TSLP measured after irradiation with 0, 20 and 50 mJ/cm^2^ UVB (*p < 0.05, ** *p* < 0.005). Data are presented as means ± standard deviation. IL-4, interleukin-4; poly I:C, polyinosinic:polycytidylic acid; siRNA, small interfering RNA; UVB, ultraviolet B.

After 6 and 24 h of poly I:C and IL-4 treatment, the mRNA expression level of 11β-HSD1 tended to decrease in siRNA-transfected NHEKs (Fig. 1a). Moreover, the cortisol concentration in the culture medium of siRNA-transfected cells was significantly lower after 6 h of treatment (Fig. 1b). Therefore, 11β-HSD1 siRNA transfection knocked down 11β-HSD1 expression and 11β-HSD1 was responsible for the regulation of cortisol production. Both 6 h and 24 h after incubation, stimulation of TSLP production by polyinosinic:polycytidylic acid (poly I:C) and interleukin-4 (IL-4)levels were higher in siRNA-transfected cells (Fig. 1c). UVB irradiation induced TSLP expression in human keratinocytes via stabilization of hypoxia-inducible factor-1α (HIF-1α)^34^. The expression of TSLP mRNA was increased in siRNA-transfected NHEKs, and the difference was significant at 100 mJ/cm^2^ UVB (Fig. 1d). TSLP levels produced by siRNA-transfected cells were significantly higher than those produced by the control cells after irradiation with 0, 20, and 50 mJ/cm^2^ UVB (Fig. 1e).

### Topical application of selective 11β-HSD1 inhibitor aggravates phenotypes inoxazolone-treated mice

**Figure 2:**
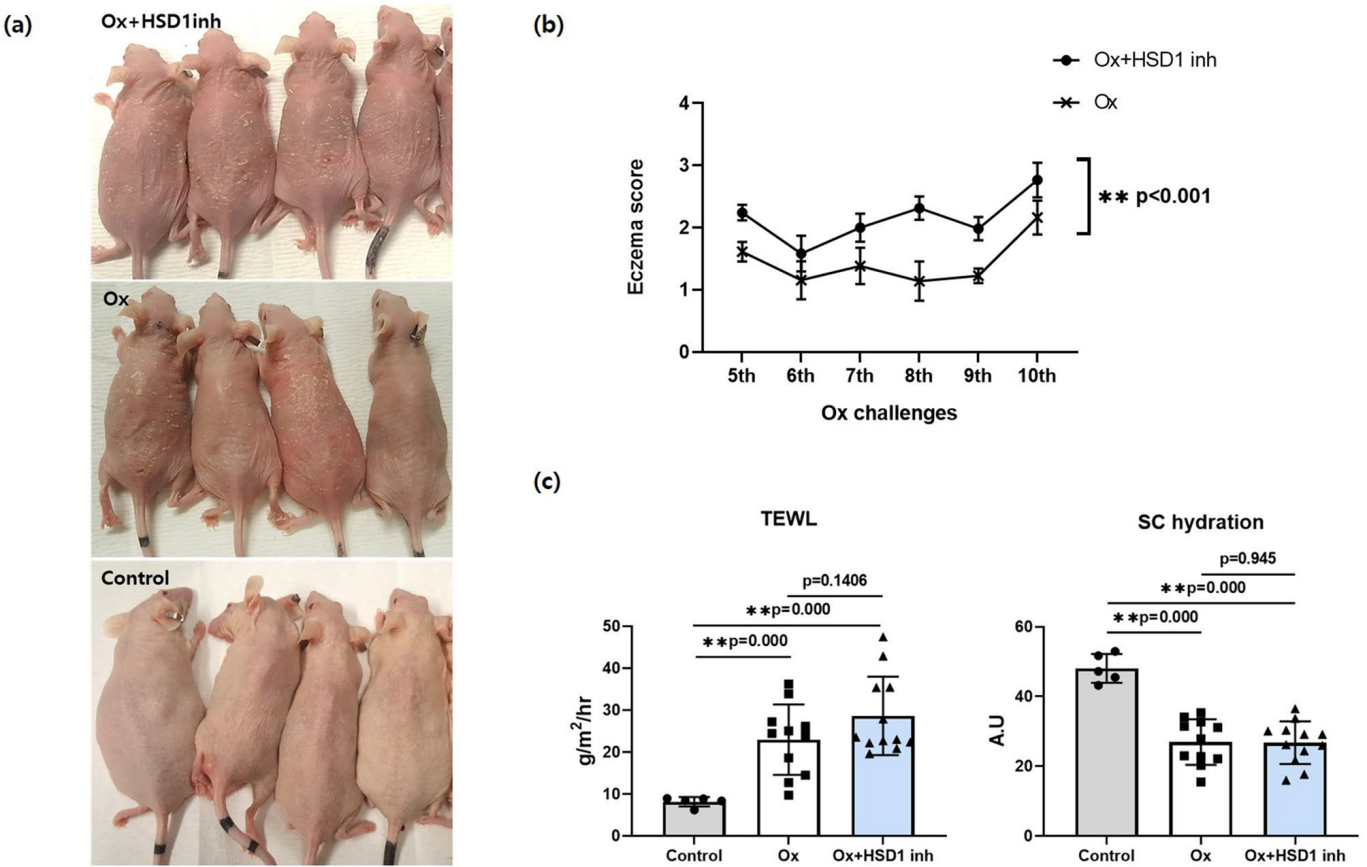
Effect of topical selective 11β-HSD1 inhibitor on oxazolone-evoked AD. (**a**) Gross photograph of control (n = 5), and Ox-induced female hairless (hr/hr) mice with topically applied vehicle (n = 7) and 11β-HSD1 inhibitor (n = 7) taken before sacrifice (at 26 days). (**b**) Eczema scores of Ox-induced mice treated with 11β-HSD1 inhibitor and vehicle at challenges 5–10. (**c**) TEWL and SC hydration in control and Ox-induced mice with vehicle and 11β-HSD1 inhibitor before sacrifice (at 26 days) (*p < 0.05, **p < 0.005). Data are presented as means ± standard deviation. *HSD1 inh* 11β-HSD1 inhibitor, *Ox* oxazolone, *SC* stratum corneum, *TEWL* transepidermal water loss.

Oxazolone (Ox)-treated mice developed typical AD lesions. Mice treated topically with 11β-HSD1 inhibitor displayed worse eczema lesions than the vehicle-treated group (Fig. 2a). The eczema score of the 11β-HSD1 inhibitor-treated group was significantly higher than that of the vehicle-treated group (Fig. 2b). Transepidermal water loss (TEWL) of both Ox-AD mouse groups was significantly increased compared to that of the control group. The 11β-HSD1 inhibitor-treated group displayed a higher (but not statistically significant) TEWL than the vehicle-treated group. SC hydration of Ox-treated mice was significantly lower than that of control mice, but there was no difference between the vehicle- and the 11β-HSD1 inhibitor-treated groups (Fig. 2c).

### Serum inflammatory cytokines tend to increase in Ox-AD mice

**Figure 3:**
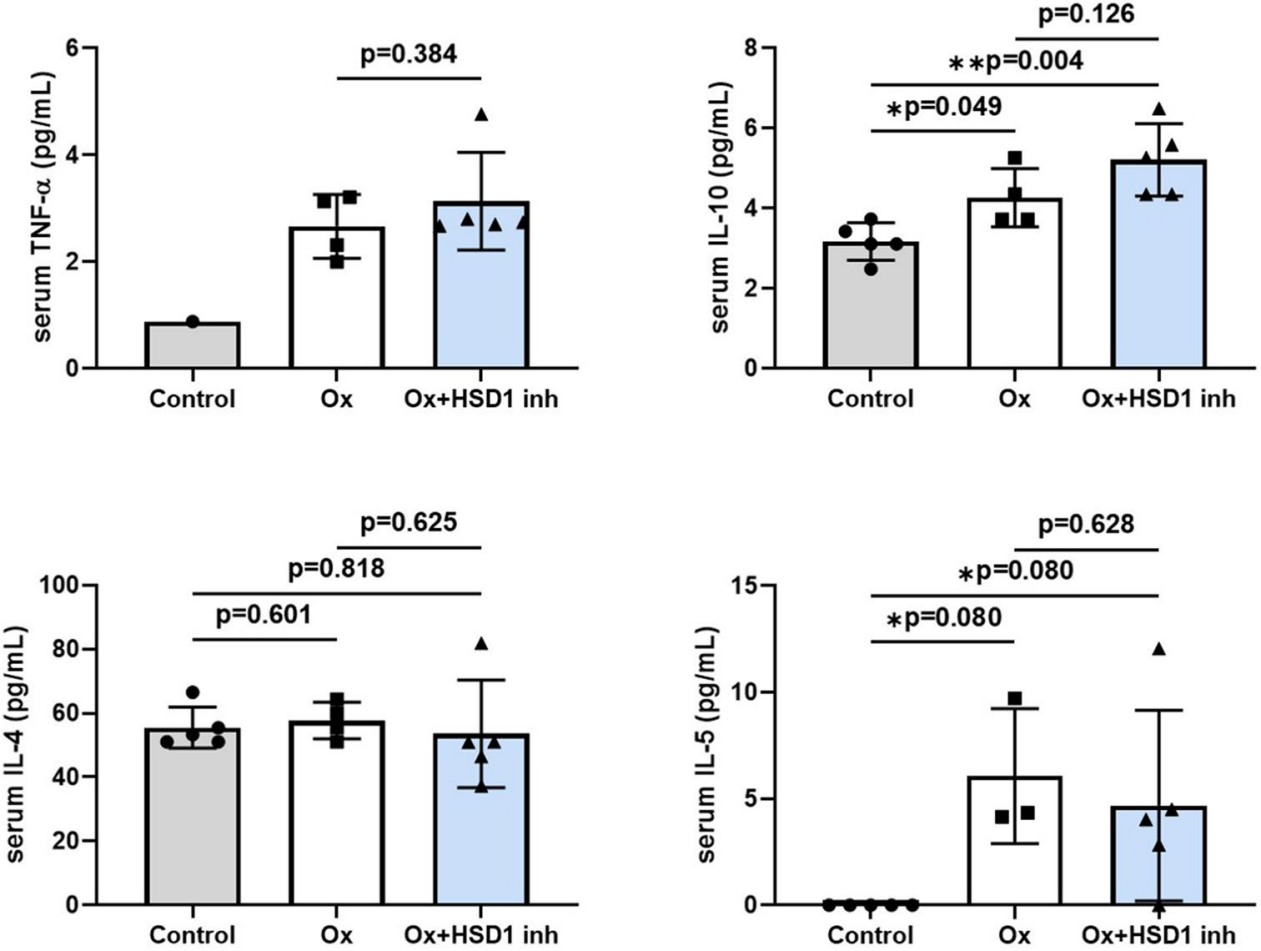
Serum inflammatory cytokines tend to be elevated in Ox-AD mice. Serum TNF-α, IL-10, IL-4 and IL-5 values determined by ELISA in control and Ox-induced mice with vehicle and 11β-HSD1 inhibitor (*p < 0.05). Data are presented as means ± standard deviation. *HSD1 inh* 11β-HSD1 inhibitor, *Ox* oxazolone.

We measured the levels of various inflammatory cytokines in mouse sera using ELISA (Fig. 3). Tumor necrosis factor-alpha (TNFα) level was increased in the Ox-AD group compared to that in the control group, but there was no difference between the 11β-HSD1 inhibitor-treated Ox-AD group and the vehicle-treated Ox-AD group. Serum IL-10 levels of the Ox-AD groups were higher than those of the control mice, and mice treated with 11β-HSD1 inhibitor showed higher serum IL-10 levels than mice from the vehicle group. Serum levels of IL-4 and IL-5 Th2 cytokines tended to be higher in Ox-AD mice. However, no significant difference was evident between the 11β-HSD1 inhibitor-treated and the vehicle-treated groups.

### Th2 cells are increased in the skin of mice treated with 11β-HSD1 inhibitor

We compared the percentage of regulatory T (Treg) cells that expressed foxp3 and CD25 among CD4-gated cells, and the percentage of Th2 cells that expressed IL-4 among CD3 and CD4-gated cells. The percentages of Treg cells and Th2 cells among splenic cells did not significantly differ between mice treated with the 11β-HSD1 inhibitor and the vehicle. The percentage of Th2 cells in skin cells tended to be higher in the 11β-HSD1 inhibitor-treated mouse group, although not significantly. Skin Treg cells did not show any significant difference between the two groups (Supplementary Fig. 1).

### Epidermal 11β-HSD1 expression is significantly increased in Ox-AD mice

**Figure 4:**
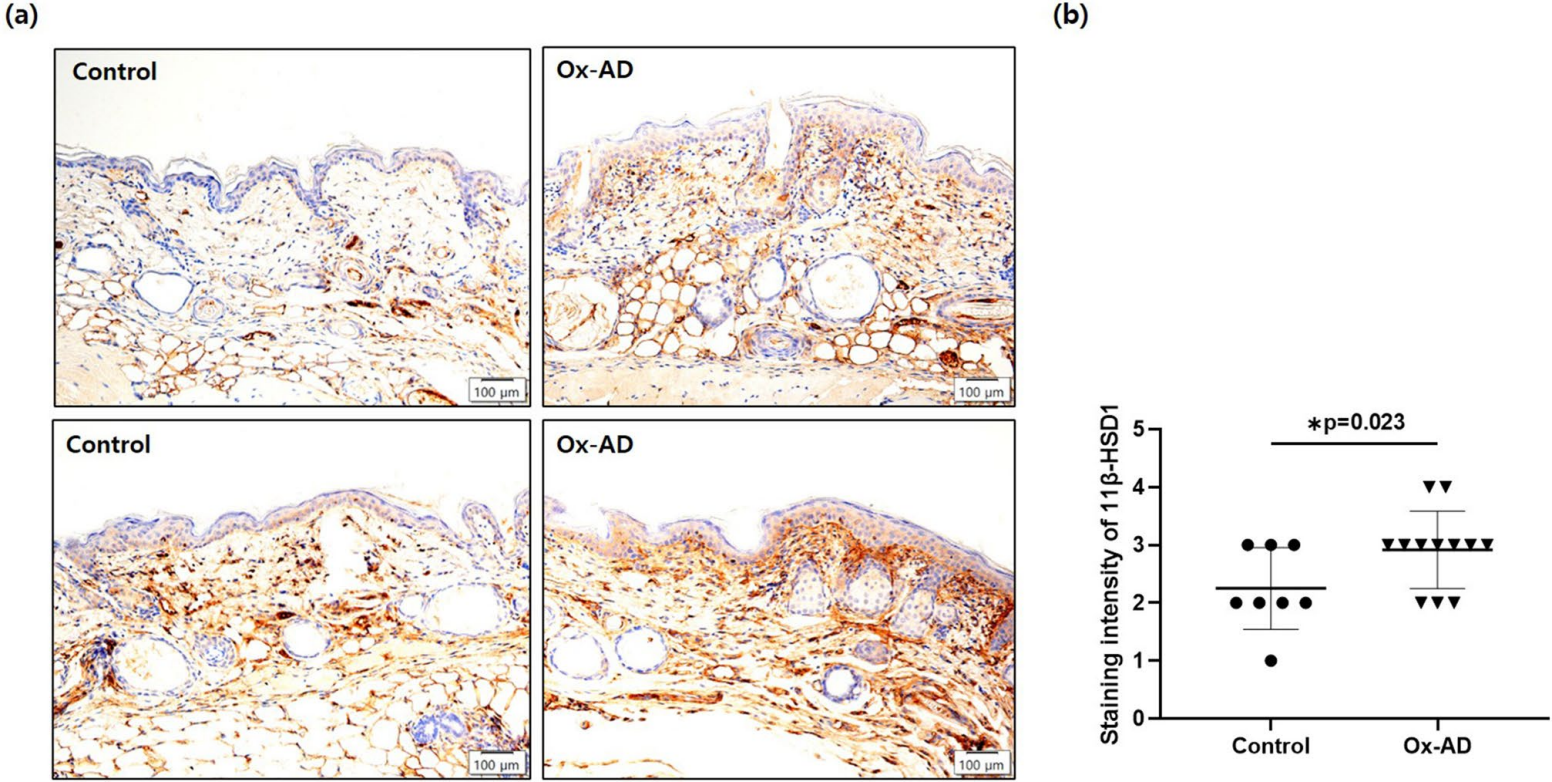
Epidermal 11β-HSD1 expression is significantly increased in Ox-AD mice. (**a**) Immunohistochemical staining and (**b**) staining intensity of 11β-HSD1 in the epidermis of control and Ox-AD mice (scale bar = 100 μm). Staining intensity of 11β-HSD1 in the epidermis was graded as 0 (none), 1 (very weak), 2 (weak), 3 (moderate), 4 (strong), and 5 (very strong). Mean scores were compared between controls and Ox-AD mice (*p < 0.05). Data are presented as means ± standard deviation. *Ox-AD* oxazolone-induced atopic dermatitis.

The staining intensity of 11β-HSD1 was significantly higher in the epidermis of Ox-AD mice than in control mice (Fig. 4a). Semi-quantitative IHC scores (from 0 to 5) of Ox-AD mice were also significantly higher than control mice (*p* = 0.023; Fig. 4b).

### Oxazolone worsened eczema in HSD11B1 KO mice compared to that in WT mice

**Figure 5:**
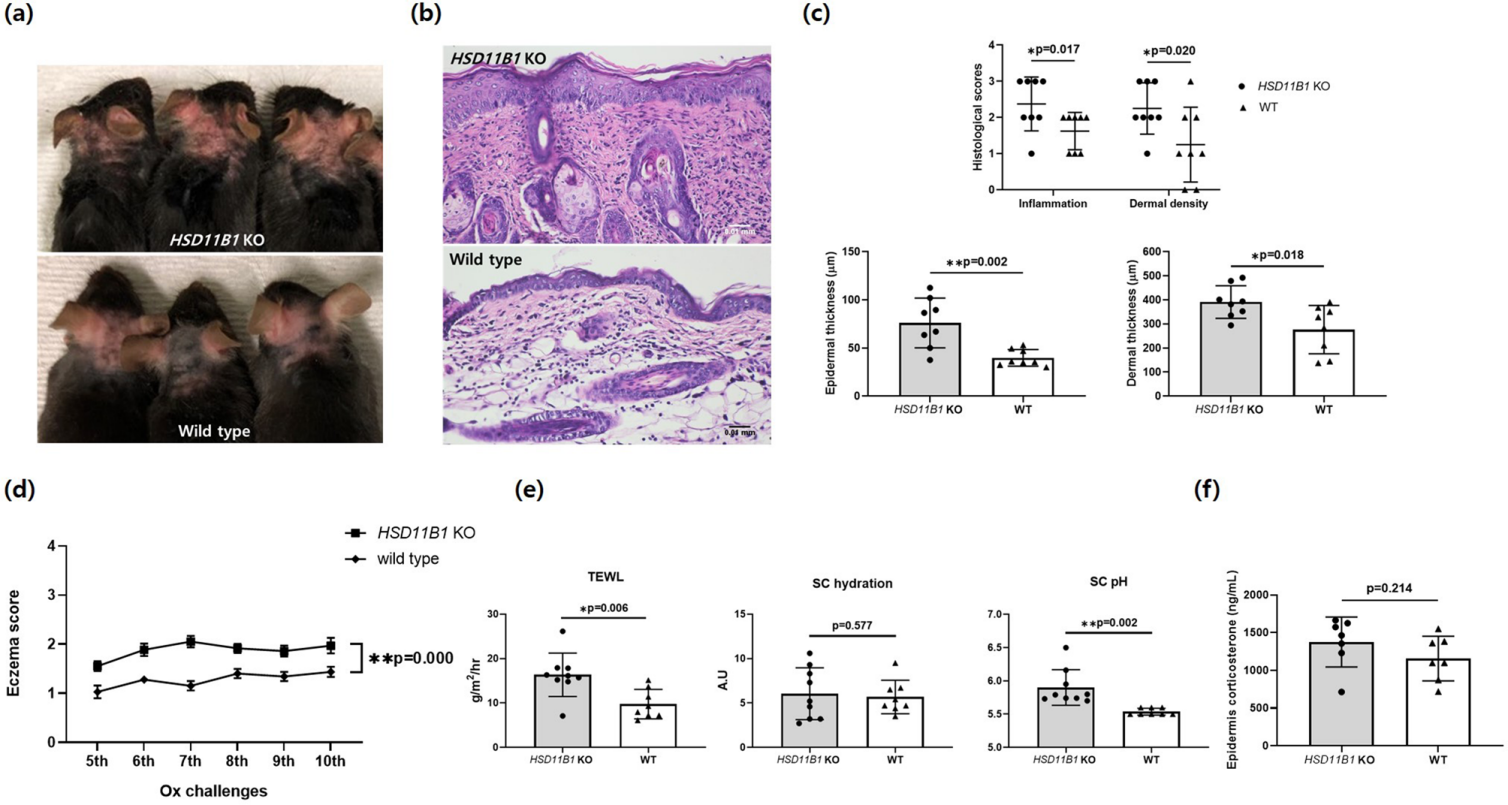
Oxazolone-induced atopic dermatitis in *HSD11B1* KO and WT mice. (**a**) Gross photographs of skin lesions in global *HSD11B1* KO mice with AD induced by Ox and WT mice (n = 8/group) taken before sacrifice (at 26 days). (**b**) Macroscopic appearance of skin sections of dorsal lesional surface. Scale bar, 10 µm. (**c**) Histological scores for dermal inflammation and density measured as epidermal and dermal thickness. (**d**) Eczema scores for *HSD11B1* KO and WT mice at challenges 5–10. (**e**) TEWL, SC pH and SC hydration measured before sacrifice (at 26th day). (**f**) Corticosterone levels in epidermis of KO and WT mice measured by ELISA (*p < 0.05, ** *p* < 0.005). Data are presented as means ± standard deviation. *KO* knock out, *SC* stratum corneum, *TEWL* transepidermal water loss.

Skin lesions were more severe after 10 Ox challenges in mice with global HSD11B1 KO than in WT mice (Fig. 5a). The pathological features of both Ox-treated groups were characterized by marked epidermal hyperplasia with spongiosis, cellular infiltrates of mononuclear and polymorphonuclear cells, and dermal thickening, which is characteristic of AD (Fig. 5b). However, atopic inflammation was worse in KO mice due to the epidermis and dermis being significantly thickened. Histological scores for dermal inflammation and collagen density were also significantly higher in the KO than in the WT mice (Fig. 5c). Eczema scores were significantly higher in the HSD11B1 KO mice (Fig. 5d). The TEWL and pH of the SC were significantly higher in the HSD11B1 KO than in the WT mice, but SC hydration did not significantly differ between the groups (Fig. 5e). Corticosterone levels were higher in the KO than in the WT mice, although the difference was not significant (Fig. 5f).

### Inflammatory cytokine expression is increased in skin of HSD11B1 KO mice

**Figure 6:**
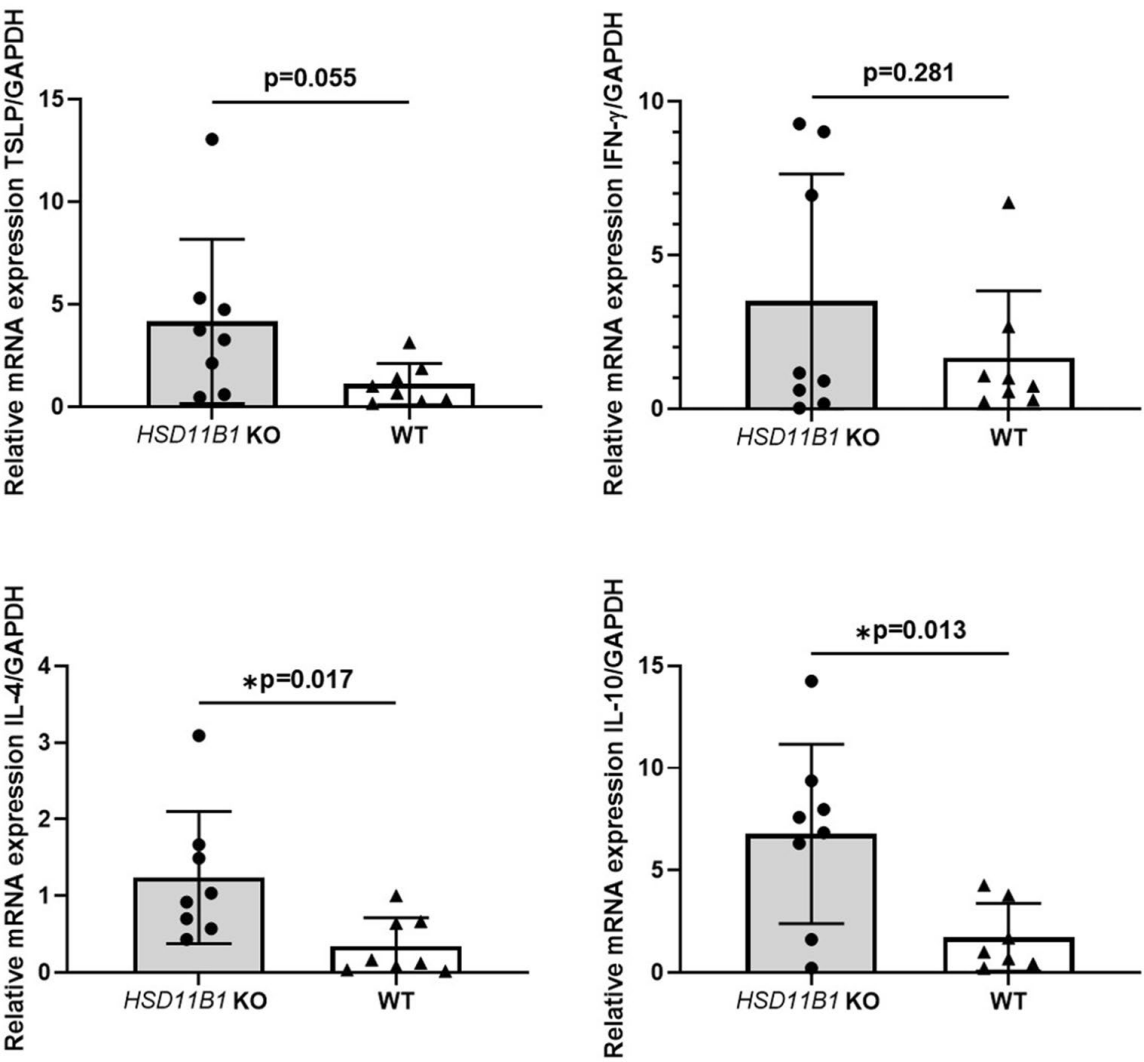
Increased inflammatory cytokine expression in skin of *HSD11B1* KO mice. Expression of TSLP, IFN-γ, IL-4 and IL-10 in epidermis of *HSD11B1* KO and WT mice analyzed using qPCR (*p < 0.05). Data are presented as means ± standard deviation. KO, knockout; WT, wild type.

The mRNA expression levels of TSLP, IL-4, and IL-10, which are crucial cytokines in AD, were increased in the epidermis of KO mice compared to the expression levels in WT mice. IL-4 and IL-10 levels were significantly higher in the KO group (p = 0.017 and 0.013, respectively). The mRNA expression of interferon-gamma (IFN-γ), an immunomodulatory cytokine, was also increased in KO mice, but the difference did not reach statistical significance (Fig. 6).

### Epidermal 11β-HSD1 expression is decreased in the lesional skin of patients with AD

**Figure 7:**
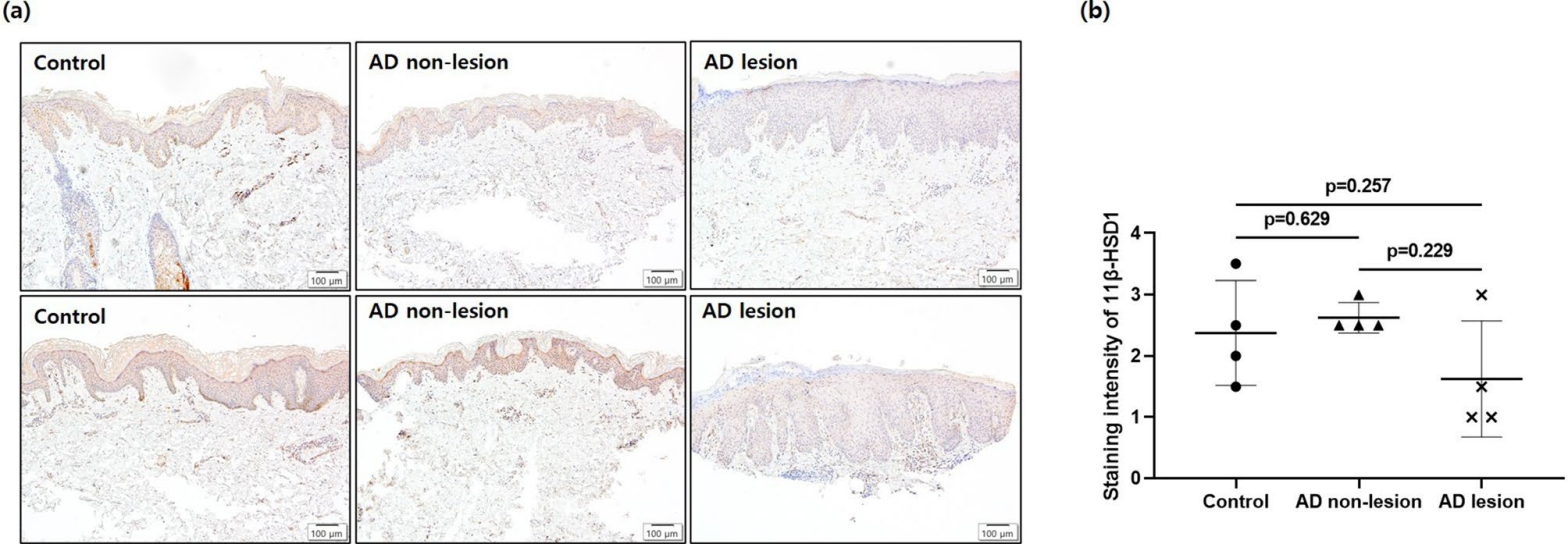
Epidermal 11β-HSD1 expression in the stratum corneum decreased in the lesional skin of patients with AD. (**a**) Immunohistochemical staining and (**b**) staining intensity of 11β-HSD1 in epidermis of lesions and non-lesions of four patients with AD and four individuals without AD (scale bar = 100 μm). Staining intensity of 11β-HSD1 in the epidermis was graded as 0 (none), 1 (very weak), 2 (weak), 3 (moderate), 4 (strong), and 5 (very strong). Mean scores were compared between controls, lesions, and non-lesions of patients with AD. Data are presented as means ± standard deviation. *AD* atopic dermatitis.

The staining intensity of 11β-HSD1 was significantly lower in the epidermis of skin lesions of patients with AD than in non-lesional skin and healthy control skin (Fig. 7a). Semi-quantitative IHC scores showed no significant difference between control and non-lesional skin of patients with AD. Lesions of patients with AD tended to have lower staining intensity than skin of healthy controls and non-lesions of patients with AD, but failed to show statistical significance (Fig. 7b).

## Discussion

Human skin functions as an independent steroidogenic organ that maintains homeostasis against external stress^35,36^. The role of 11β-HSD1 in inflammation has been studied. Inhibition of 11β-HSD1 in murine macrophages treated with lipopolysaccharide suppresses pro-inflammatory cytokine expression^37^. Moreover, inhibiting 11β-HSD1 reduces the TNFα-induced expression of IL-6, whereas 11β-HSD1 overexpression further augments IL-6 expression^38^. Knockdown of 11β-HSD1 in NHEK by siRNA transfection abrogates the induction of IL-6 and IL-8 by IL-1β and TNFα^4^. These results suggest that 11β-HSD1 expression is needed to produce inflammatory cytokines.

On the contrary, 11β-HSD1 plays a protective role in models of inflammation in vivo. 11β-HSD1-deficient K/B × N serum-induced arthritis model mice showed earlier onset and slower resolution of inflammation^39^. When contact dermatitis was induced in mice using hapten, keratinocyte-specific 11β-HSD1 KO mice showed enhanced inflammation compared to WT mice^24^. These inconsistent results may reflect the complexity of systemic and local GC production affected by a de novo pathway and 11β-HSD1 action.

Two distinct 11β-HSDs and four distinct 17β-HSDs are known in mammals. The preference of the two 11β-HSDs and four 17β-HSDs for catalyzing oxidation or reduction of substrates has physiological benefits. According to Baker et al., the selective expression of different enzymes at different times in different tissues can control the concentration of active GCs or estrogens and androgens, providing a mechanism for regulating the actions of these hormones and maintaining normal physiology^40^. Phylogeny suggests that evolution of these enzymes from ancestor HSD may have occurred during the transition of vertebrates from ocean to land due to selective pressures from the colonization of land. 11β-HSD1 is most abundant in the liver and adipose tissue, but it is also widely expressed in the epidermis and dermis. In contrast, 11β-HSD2 shows lower expression in the skin^41^. As the skin is the outermost organ of the body and is in constant contact with the environment, inflammation frequently occurs and keratinocytes differentiate repeatedly. Due to the nature of the skin, 11β-HSD1 was highly conserved in the skin through human evolution and served as an “endogenous pharmacy.” However, complications, such as skin aging and inhibition of wound healing, appear when the enzyme is overexpressed as a result of UV exposure and endogenous aging.

We found that 11β-HSD1 expression was increased in the epidermis of flaky tail mouse models of congenital AD that spontaneously develop AD lesions. Sensitizing the skin of flaky tail mice with a mite allergen further increased 11β-HSD1 expression (data not published). Thus, we concluded that 11β-HSD1 action is important for dampening inflammation in the skin through GC activation, and that the reduced activity of this enzyme might promote the development of inflammatory skin diseases, including AD. Therefore, we speculated that inhibition of 11β-HSD1 decreases the levels of active cortisol in the skin and aggravates AD severity in the mouse model of Ox-induced AD.

A study to determine whether *11β-HSD1* knockdown in keratinocytes affects the production of TSLP that functions as a master switch for the Th2 immune response in AD^42^ found significantly higher TSLP production after stimulation with poly I:C and IL-4 in siRNA-transfected NHEK. These results agree with later findings^33^. Irradiation with UVB induces TSLP expression in human keratinocytes^34^. The present study found that siRNA transfection increased the amount of TSLP induced by UVB irradiation. Although we found that siRNA transfection almost completely inhibited 11β-HSD1 expression, *11β-HSD1* KO only partly inhibited cortisol production by keratinocytes (Fig. 2b). This is because the skin can synthesize extra-adrenal glucocorticoid. Slominski et al. found that skin and skin cells express the genes and proteins required to activate the cytochrome P450scc system, indicating that skin can metabolize glucocorticoid from cholesterol^43^.

The local inhibition of 11β-HSD1 in the skin of Ox-AD hairless mice should aggravate the severity of atopic inflammation by suppressing GC activation. Eczema lesions, developed in the skin of hairless mice after four applications of Ox and worsened according to eczema scores in mice that were topically treated with an 11β-HSD1 inhibitor. The skin permeability barrier function, indicated by basal TEWL and SC hydration, was disrupted in Ox-AD mice compared with that in control mice, but those treated with vehicle and with the 11β-HSD1 inhibitor did not significantly differ.

There are no reported protocols regarding the concentration of topical 11β-HSD1 inhibitor (385581). However, in a report published in 2014, Terao and colleagues dissolved 385581 in DMSO (10 μM) for subcutaneous injection daily for 2 days^44^. They also used 385581 (50 μM) dissolved in acetone:olive oil (1:1) applied topically every day for 5 days and 385581 dissolved in PBS (10 μM) to apply to skin wounds in a report published in 2011^18^. Neither report indicated a specific reference or consensus for their concentration choices. In our study, we chose a concentration of 100 μM of 385581 dissolved in DMSO, which is easily accessible as a vehicle in the laboratory, and 100 μL was applied to the skin twice a day. The results of our experiment were as expected from our hypothesis, suggesting that this concentration was effective.

Serum levels of inflammatory cytokines were higher in Ox-AD mice than in control mice, and even more so in mice with localized 11β-HSD1-inhibition, although the differences did not reach statistical significance. IL-10 induces Th2 but also has anti-inflammatory effects, and is increased in skin lesions in patients with AD^45^. Decreased IL-10 levels are associated with AD flares^46^. IL-10 forms heterodimers and binds to the IL-10 receptor to activate the Janus kinase-signal transducer and activator of transcription signaling pathway, notably Janus kinase 1, tyrosine kinase 2, and signal transducer and activator of transcription 3 and 1^47^. Its anti-inflammatory effects are mediated by inhibition of IFN-γ and IL-2 production by Th1 cells and inhibition of IL-4 and IL-5 production by Th2 cells through interference with B7/CD28-dependent signals^48^. The present study showed increased IL-10 levels in Ox-AD mice and higher mean IL-10 levels in mice that were topically treated with 11β-HSD1 inhibitor compared to vehicle-treated mice; however, the difference was not statistically significant. These results implied that although locally inhibited GC activation worsened eczema, it was insufficient to cause systemic effects. On the other hand, compared to WT mice, *HSD11B1* KO mice had significantly higher levels of IL-10 in the skin. It would have been useful if genes or proteins associated with IL-10 were evaluated to determine the consequences of increased IL-10 on AD inflammation, but this could not be done due to a lack of samples.

Fluorescence-activated cell sorting (FACS) showed that the number of Th2 cells tended to increase in the skin of mice with localized 11β-HSD1 inhibition. However, the numbers of foxp3 + Treg and splenic cells in skin did not significantly differ. Treg cells are immuneregulators that diminish excessive immune responses, including those of harmful Th2 cell^49^. Because splenic cells represent systemic inflammation, topical 11β-HSD1 inhibition might have weakened Th2 inflammation exclusively in the skin by blocking GC conversion to its active form. This may share meaning with the cytokine analysis in the skin, although we could not measure cytokine levels in the skin of mice due to a lack of skin samples.

We assessed the impact of systemic 11β-HSD1 inhibition on the emergence of AD lesions in global *HSD11B1* KO mice in vivo. Others have validated the role of 11β-HSD1 in KO mice. One study describes the phenotypes of mice with targeted *11β-HSD1* and *11β-HSD2* deletions. Serum corticosterone is hypersecreted and the adrenal glands are hypertrophied in *HSD11B1* KO mice due to the absence of normal negative feedback and failure of the HPA axis to compensate^50^. Fasting glucose levels were similar between the KO and WT mice, but the responses of gluconeogenic enzymes were attenuated after starvation in the KO mice and they were resistant to hyperglycemia when stressed. Others have noted a discrepancy between secretion of local and systemic corticosterone in these KO mice, which has hindered clarification of the role of 11β-HSD1 in dermal collagen metabolism^44^. In contrast, one study found that HPA axis abnormalities in response to 11β-HSD1 are strain-dependent^51^, because congenic *HSD1* KO mice on a C57Bl/6J background have normal basal plasma corticosterone and ACTH concentrations, in contrast to 129/MF1 mice that are null for *11β-HSD1*. Others have found that age-induced dermal atrophy is reversed and that the collagen biosynthesis gene is expressed in *HSD11B1* KO mice^23^. Results obtained with keratinocyte-specific *HSD11B1* KO mice were consistent with the notion that corticosterone activation by 11β-HSD1 in keratinocytes represses local inflammation^22,24^; this rules out the impact of a compensatory increase in serum corticosterone in global KO mice. Moreover, serum corticosterone concentrations did not differ between WT and keratinocyte-specific KO mice.

We confirmed that the systemic inhibition of 11β-HSD1 significantly worsened AD evoked by Ox. These results were also supported by the histological features of a thickened epidermis and dermis, as well as higher scores for dermal inflammation and density in KO mice. Skin barrier permeability functions were also decreased in the KO mice. TEWL and SC pH was higher in KO mice. Elevated SC pH increases activity of kallikreins, which contributes to over degradation of corneodesmosomes followed by weakening of SC integrity and cohesion. It also impairs lipid processing and disrupts the permeability barrier of the epidermis. Therefore, antigen uptake increases through the disrupted skin barrier, which activates Langerhans cells, and then PAR-2 and TSLP are increased in the blood as well as the skin, contributing to AD-like inflammation^52,53^.

The principle cytokines involved in the pathogenesis of AD were increased in the epidermis of KO mice compared with WT mice. In contrast, corticosterone levels tended to be higher in the epidermis of KO mice. This might have been due to excessive corticosterone production by the central HPA axis or local production by the peripheral HPA axis in the skin to compensate for insufficient peripheral corticosterone. We investigated whether *HSD11B1* KO mice have functional circadian rhythms by measuring serum corticosterone (n = 1 per time point). Corticosterone values peaked at 1 the 5:00 and 18:30 hours and the reached at rough at 09:00 and 21:00 hours. The overall trend was similar to previous findings but differed somewhat in terms of peak timing (Supplementary Fig. 3). The range of serum corticosterone values was similar to that of previous findings^54^. We collected blood and skin samples at 09:00 hours which could be a disadvantage, but four investigators simultaneously sampled the mice, therefore it could be assumed that results were not influenced by the difference in time of sample collection.

The intensity of IHC staining for 11β-HSD1 in the epidermis showed the opposite results between murine AD models and human subjects. AD mice induced with Ox application exhibited an increased concentration of 11β-HSD1 in the epidermis compared to the controls. On the contrary, lesional skin of patients with AD showed the lowest 11β-HSD1 intensity compared to non-lesions of patients with AD and healthy control subjects, although the *p*-value did not reach statistical significance. Our results are similar to those of Terao et al., who reported that the expression of 11β-HSD1 increased in the epidermis of murine models after exposure of the models to 0.2% Oxa^24^; however, the expression of 11β-HSD1 is significantly decreased in the epidermis of patients with AD^33^. The discrepancy between murine and human subjects has yet to be investigated, but we propose the possibility that although short-term inflammation enhances 11β-HSD1 levels to suppress the inflammation, consistent long-term production of Th2 type cytokines may exert an inhibitory effect on the activity of 11β-HSD1 in the skin.

Our study results in vitro and in vivo confirmed that 11β-HSD1 plays a role in the development of AD. As a pathogenic mechanism of AD, disruption of the skin permeability barrier leads to the increase of various inflammatory cytokine, evoking chronic inflammation in the skin^14,55^. In addition, activation of 11β-HSD1 in the skin protects against atopic inflammation by increasing the local availability of active GC, which are potent immuno-suppressants. Although control of systemic cortisol/corticosterone by the HPA axis is important for anti-inflammation, the de novo pathway and local control by 11β-HSD1 are also important in the skin. We have identified that inhibition of 11β-HSD1 activity alone aggravates AD inflammation irrespective of serum CS levels, and also verified that 11β-HSD1 activity is decreased in the skin of AD patients. In conclusion, insufficient 11β-HSD1 production or activity could function in the intricate pathophysiology of AD. If so, using methods that control local activity of 11β-HSD1 in the skin to treat atopic dermatitis could provide further insight into novel therapeutic approaches that prevent side effects from steroids that control systemic corticosterone.

## Methods

### Studies in vitro

#### Transfection with siRNA

We transfected NHEKs with 50 nM 11β-HSD1 siRNA or control siRNA (Bioneer, Daejeon, Korea) using a mixture of Opti-MEM and Lipofectamine RNAiMAX reagent (Invitrogen, Carlsbad, CA, USA). The culture medium was replaced after six hours and the cells were used for experiments at 48 h after transfection. Detailed information about siRNA transfection, poly I:C and IL-4 treatment, UVB irradiation, qPCR, and ELISA is provided in the online Supplementary Materials and Methods.

### Studies in vivo

#### Mouse experiments

The Institutional Animal Care and Use Committee at Yonsei University Wonju College of Medicine approved all animal procedures (YWC-150303-1). All animal handling procedures and human involvement methods were carried out in accordance with relevant guidelines and regulations. Eight-week-old female hairless mice (hr/hr) were obtained from OrientBio (Seongnam, Korea). Oxazolone was purchased from Sigma-Aldrich Co. (St. Louis, MO, USA). The selective 11β-HSD1 inhibitor, 385581 (Merck & Co., Kenilworth, NJ, USA), was dissolved in dimethyl sulfoxide (DMSO; 100 μM) vehicle for topical application.

The control group consisted of five mice, and the Ox-treated groups consisted of seven mice, each. Atopic dermatitis-like lesions were induced by the topical application of Ox (Supplementary Fig. 2). The mice were sensitized with a single application of 1% Ox (60 μL). One week later, 0.1% Ox (60 μL) was topically applied every other day for 20 days. We concurrently applied 100 μL of 385581 twice daily to both sides of half of the mice (seven) with induced AD and DMSO vehicle to the other half (seven mice). The control group was similarly treated with acetone. Eczema was scored at every challenge, and gross AD lesions were photographed on the day the mice were sacrificed. The eczema score was defined as the average of individual scores for symptoms of erythema, edema, and lichenification, and graded as 0 (none), 1 (mild), 2 (moderate) or 3 (severe)^56^. Excoriation was excluded, as it is difficult to evaluate in mice. We measured TEWL on the dorsal skin of mice using a TEWA meter (Courage + Khazaka Electronics GmbH., Köln, Germany) and SC hydration using a Corneometer (Courage + Khazaka) before sacrifice (at 26 days). Dorsal skin, spleen and blood samples were collected from the mice post mortem.

#### Histological analysis

Detailed descriptions are provided in the Supplementary Materials and Methods, which are available online.

#### Enzyme-linked immunosorbent assay (ELISA)

Detailed descriptions are provided in the online Supplementary Materials and Methods.

#### Flow cytometry

Detailed descriptions are provided in the online Supplementary Materials and Methods.

#### HSD11B1 KO mice

Mouse embryos with global HSD11B1 KO were supplied by Professor Gareth G. Lavery of the Molecular Metabolism Centre for Endocrinology, Diabetes, and Metabolism, School of Clinical and Experimental Medicine, Institute of Biomedical Research University of Birmingham, UK. We generated and injected HSD11B1 KO gene clones into C57BL/6 blastocysts as described^57^. The embryos were transferred into pseudo-pregnant C57BL/6 female mice and heterozygous mice were generated. Germline transmission of the KO allele was confirmed by PCR genotyping, then homozygous HSD11B1 KO mice were obtained after several breeding cycles Details of the genotyping are provided in the online Supplementary Materials and Methods.

#### Experiments using HSD11B1 KO mice

Oxazolone was applied as described above to induce AD-like lesions in 11- to 20-week-old KO and WT mice (n = 8 each). One day after the 10^th^ Ox challenge, eczema was scored and photographed in each mouse, then TEWL, SC hydration, and SC pH were measured before sacrifice at 26 days. Dorsal and ear skin, and blood samples were taken post mortem.

#### Histological analysis

Detailed descriptions are provided in the online Supplementary Materials and Methods.

#### Quantitative polymerase chain reaction (qPCR)

A detailed description of the qPCR is provided in the online Supplementary Materials and Methods.

#### Studies on human samples

Detailed descriptions are provided in Supplementary Materials and Methods, which are available online.

### Statistical analyses

Data in graphs are expressed as means ± standard deviation. Differences between groups were analyzed by Student *t* tests using GraphPad Prism (GraphPad Software, La Jolla, CA, USA). Differences between treated groups at each time point were determined using two-way analysis of variance (ANOVA) followed by Bonferroni-Dunn tests. Mann–Whitney test was used to compare the staining intensities of human skin samples. Values with p < 0.05 were considered statistically significant.

## Supplementary information

Supplementary Information.

## Data Availability

No datasets were generated or analyzed during the current study.
