# Supplementary material for: Role of 11β-hydroxysteroid dehydrogenase type 1 in the development of atopic dermatitis
Source: Sci Rep. 2020 Nov 19;10:20237. doi: 10.1038/s41598-020-77281-x (PMC7678864; doi:10.1038/s41598-020-77281-x)
Supplement: Supplementary file 1 — Supplementary Information. [file 41598_2020_77281_MOESM1_ESM.docx]

**Role of 11β-hydroxysteroid dehydrogenase type 1 in the development of atopic dermatitis**

Noo Ri Lee^1^, Beom Jun Kim^1^, Chung Hyeok Lee^1^, Young Bin Lee^1^, Solam Lee^1^, Hyun Jee Hwang^1^, Eunjung Kim^1^, Sung Hee Kim^2^, Min-Geol Lee^2^, Sang Eun Lee^3^, Gareth G. Lavery^4^, Eung Ho Choi^1^

^1^Department of Dermatology, Yonsei University Wonju College of Medicine, Wonju, Korea

^2^Department of Dermatology, Severance Hospital, Cutaneous Biology Research Institute, Yonsei University College of Medicine, Seoul, Korea

^3^Department of Dermatology, Cutaneous Biology Research Institute, Yonsei University College of Medicine, Gangnam Severance Hospital, Seoul, Korea

^4^Institute of Metabolism and Systems Research, College of Medical and Dental Sciences, University of Birmingham, Birmingham B15 2TT, United Kingdom

**Supplementary Methods**

**Study *in vitro***

**Small interfering RNA transfection**

The Institutional Review Board at Yonsei University Wonju College of Medicine approved this procedure (CR317026). We obtained NHEK from the foreskins of 12-year-old boys who were circumcised in the Urology Department. Skin tissues were sectioned, rinsed with phosphate buffered saline, and cultured at an air-medium interface in Dulbecco’s modified Eagle’s medium (DMEM) supplemented with 10% fetal calf serum, 1 mg/mL ciprofloxacin and 200 mM L-glutamine at 37°C in a 5% CO_2_ atmosphere. The NHEK (50,000/mL) were cultured in EpiLife™ medium (Thermo Fisher Scientific, Waltham, MA, USA) supplemented with an antibiotic-antimycotic and human keratinocyte growth supplement (Thermo Fisher Scientific) at 37°C in a 5% CO_2_ atmosphere. One day before transfection, the cells were seeded on plates coated with type-1 collagen.

**Poly I:C and IL-4 application**

Poly I:C is an immunostimulant that activates Toll-like receptor 3 (TLR3) and because keratinocytes express large amounts of TLR3, poly I:C stimulation provokes an inflammatory reaction. Stimulating keratinocytes with poly I:C plus IL-4 increases NF-κB activity and the NF-κB-dependent production of TSLP ^1^. We used poly I:C HMW (InvivoGen, Inc., San Diego, CA, USA) and IL-4 (R&D Systems, Minneapolis, MN, USA). Portions (180 μL) of an NHEK suspension (250,000/mL) in DMEM containing 4.5 g/L glucose, 10% (v/v) heat-inactivated fetal bovine serum (30 min at 56°C), 50 U/mL penicillin, 50 μg/mL streptomycin, 100 μg/mL Normocin, and 2 mM L-glutamine were incubated in the wells of 96-well plates for 10 min at 65°C to 79°C. The cells were cooled at room temperature (22-26°C) for 60 min then stimulated with 10 μg/mL of poly I:C HMW for six or 24 h.

**UVB irradiation**

The NHEK transfected with siRNA or control siRNA were irradiated with 0, 20, 50 of 100 mJ/cm^2^ of UVB light using a TL20W/12 RS UVB lamp (Philips, Utrecht, Netherlands) with emission at 290-315 nm.

**Quantitative polymerase chain reaction (qPCR)**

Total RNA was isolated from NHEKs using QuantiTect Reverse Transcription Kits (Qiagen, Hilden, Germany). The product was reverse-transcribed into first-strand complementary DNA (cDNA), then 11β-HSD1 expression was measured using QuantiFast SYBR Green PCR Master Mix (Qiagen) as described by the manufacturer. The following sequence-specific primers were designed: 11β-HSD1, sense: 5ʹ-TCTCCTCTCTGGCTGGGAAAG-3ʹ, antisense: 5ʹ-GAACCCATCAAAGCAAACTTG-3ʹ; TSLP, sense: 5ʹ-TAGCAATCGGCCACATTGCCT-3ʹ, antisense: 5ʹ-GAAGCGACGCCACAATCCTTG-3ʹ.

**Enzyme-linked immunosorbent assay (ELISA)**

Culture supernatants were separated by centrifugation at 1000 × *g* for 15 min at 8°C. The amount of cortisol in samples was measured using human cortisol ELISA kits (Cusabio Technology LLC., Houston, TX, USA), as described by the manufacturer. The expression of TSLP was measured using human TSLP Quantikine ELISA kits (#DTSLP0; R&D Systems).

**Mouse studies *in vivo***

**ELISA**

Debris was removed from mouse serum samples by centrifugation at 2000×*g* for 20 min. Thereafter, 75 μL of the serum sample and 75 μL of the RD6-52 calibrator diluent were mixed at a two-fold dilution. Serum levels of TNFα, IL-4, IL-5, and IL-10 were analyzed using Magnetic Luminex screening assay kits (R&D Systems) as described by the manufacturer. Mixtures of samples and antibody were incubated for 2 h at room temperature (22–26°C), then analyzed using a Luminex 100 device (Luminex, Austin, TX, USA). We evaluated the circadian rhythms of corticosterone in serum collected from one *HSD11B1* KO mouse at 09:00, 15:00, 18:30, and 21:00 h using ab100821 corticosterone ELISA kits (Abcam). Serum samples were diluted 100-fold, then ELISA was performed as described by the manufacturer.

**Histological analysis**

Skin samples were collected from 8 control mice and 12 Ox-AD mice the day after 10 Ox challenge and sectioned. The sections (4 μm thick) were deparaffinized, rehydrated, and incubated with a peroxidase‐blocking reagent (Dako REAL Peroxidase-blocking solution; Agilent Technologies, Santa Clara, CA, USA) for 10 min. Nonspecific protein binding was inhibited by incubating the samples with serum‐free protein for 10 min at room temperature (22–26°C), followed by overnight incubation with primary antibodies (diluted 1:100) against 11β-HSD1 (Cayman Chemical, Ann Arbor, MI, USA) at 4°C. The sections were then incubated with horseradish peroxidase‐conjugated secondary antibody (HRP Rabbit/Mouse Dako; Agilent Technologies, Santa Clara, CA, USA) for 1 h at room temperature. Antigen-antibody complexes were visualized after staining the sample with 3,3′-diaminobenzidine. Cell nuclei were counterstained with hematoxylin (Invitrogen, Carlsbad, CA, USA). Staining intensity was graded as 0 (none), 1 (very weak), 2 (weak), 3 (moderate), 4 (strong), or 5 (very strong).

***HSD11B1* KO mice genotyping**

We genotyped *HSD11B1* alleles using conventional PCR. The following primers were multiplexed: P1, 5ʹ-GGGAGCTTGCTTACAGCATC-3ʹ; P2, 5ʹ-CATTCTCAAGGTAGATTGAACTCTG-3ʹ; P3, 5ʹ-TCCATGCAATCAACTTCTCG-3ʹ. Primers P2 and P3 produced a band of 139 bp, indicating a WT allele. Amplicons indicating amplification between P1 and P3 were undetectable due to the distance between these primers. A P2 binding site was removed and P1 and P3 were brought into proximity in the KO allele to generate a 242-bp product with which to detect WT, heterozygotes and homozygotes.

**Histological analysis**

Skin tissue specimens were immediately placed in 10% neutral buffered formalin and processed for paraffin embedding and histological staining using hematoxylin and eosin (H&E). Four samples from each group of mice were examined using an Olympus BX51 microscope and a Canon EOS 550D camera. Images were acquired at 200× magnification. The epidermal thickness was measured from the basal cell layer to the surface horny layer using ImageJ (National Institutes of Health, Bethesda, MD) at four points in cross-sections. Dermal thickness was measured from the dermal-epidermal and dermal-subcutaneous fat junctions at four points. Dermal inflammation was scored as 0 (none), 1 (mild), 2 (moderate), or 3 (severe). Dermal collagen density was scored as 0 (low), 1 (mild), 2 (moderate) or 3 (severe).

**Quantitative PCR**

Total RNA was isolated from cultured cells and powdered tissues using QIAzol (RNeasy lipid tissue kit; Qiagen) as described by the manufacturer. Messenger RNA was quantified by real-time reverse transcription polymerase chain reactions (RT-PCR) using a 7900HT FAST Real-Time PCR System (Applied Biosystems, Franklin Lakes, NJ, USA). Primer concentrations were firstly optimized to avoid non-specific binding of primers. After the PCR, dissociation curves were analyzed to verify the specificity of the amplification products. The mRNA was normalized using glyceraldehyde 3-phosphate dehydrogenase (GAPDH), and the following sequence-specific primers were designed: GAPDH sense, 5ʹ-TTGATTTTGGAGGGATCTCG-3ʹ and antisense, 5ʹ-GAGTCAACGGATTTGGTCGT-3ʹ; IFN-γsense, 5ʹ-TCAAGTGGCATAGATGTGGAAGAA-3ʹ and antisense, 5ʹ- TGGCTCTGCAGGATTTTCATG-3ʹ; IL-4 sense, 5ʹ- ACAGGAGAAGGGACGCCAT-3ʹ and antisense, 5ʹ-GAAGCCGTACAGACGAGCTCA-3ʹ; IL-10 sense, 5ʹ-TGGCCCAGAAATCAAGGAGC-3ʹ and antisense, 5ʹ-CAGCAGACTCAATACACACT-3ʹ; TSLP sense, 5ʹ-CGACGAAATCGAGGACTGTGA-3ʹ and antisense, 5ʹ-TAGCCTGGGCAGTGGTCATT-3ʹ. The conditions for thermal cycling were as follows: denaturation for five min, followed by 45 cycles of amplification at 95°C for 15 s, annealing at 60°C for 30 s, and extension at 76°C for 30 s.

**Studies of human samples**

**Immunohistochemical (IHC) staining of 11β-HSD1**

The Institutional Review Board at Yonsei University Wonju College of Medicine approved this procedure (CR317026). Informed consent was obtained from all subjects and for subject under 18, from a parent and/or legal guardian. Lesional and non-lesional skin samples of four patients with AD were embedded in paraffin. Normal human skin samples were collected from four donors who underwent unrelated skin surgery. The samples were sectioned and the sections (4 μm thick) were deparaffinized, rehydrated, and incubated with a peroxidase‐blocking reagent (ab64218; Abcam, Cambridge, UK) for 10 min. Nonspecific protein binding was inhibited by incubating the samples with serum‐free protein for 10 min at room temperature (22–26°C), followed by an overnight incubation with primary antibodies (diluted 1:100) against 11β-HSD1 (Cayman Chemical, Ann Arbor, MI, USA and Abcam, Cambridge, UK) at 4°C. The sections were then incubated with horseradish peroxidase‐conjugated secondary antibody for 1 h at room temperature. Antigen-antibody complexes were visualized after staining the samples with 3,3′-diaminobenzidine. Cell nuclei were counterstained with hematoxylin (Invitrogen, Carlsbad, CA, USA). Staining intensity was graded as 0 (none), 1 (very weak), 2 (weak), 3 (moderate), 4 (strong), or 5 (very strong).

**Supplementary Figures**


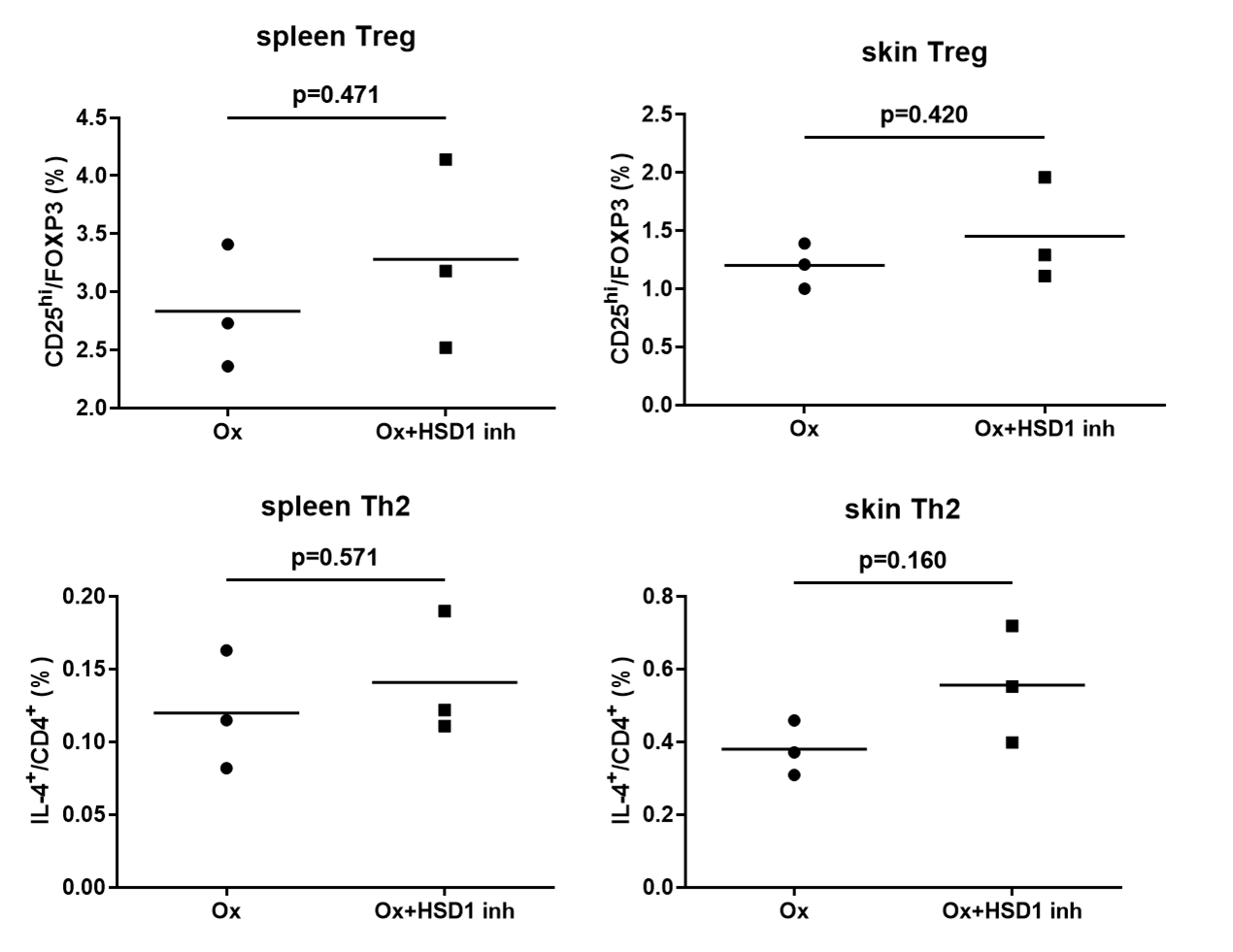


**Supplementary Figure 1. Increased numbers of Th2 cells in the skin of mice treated with 11β-HSD1 inhibitor**

Analysis of spleen and skin cells from Ox-AD mice topically treated with vehicle or 11β-HSD1 inhibitor using FACS. Graphs show populations of regulatory T (Treg) cells expressing foxp3 and CD25 among CD4 gated cells, and of Th2 cells expressing IL-4 among CD3 and CD4 gated cells. Data are presented as individual data and means. HSD1 inh, 11β-HSD1 inhibitor; Ox, oxazolone; Th2, T helper type 2 cells; Treg, regulatory T cells.


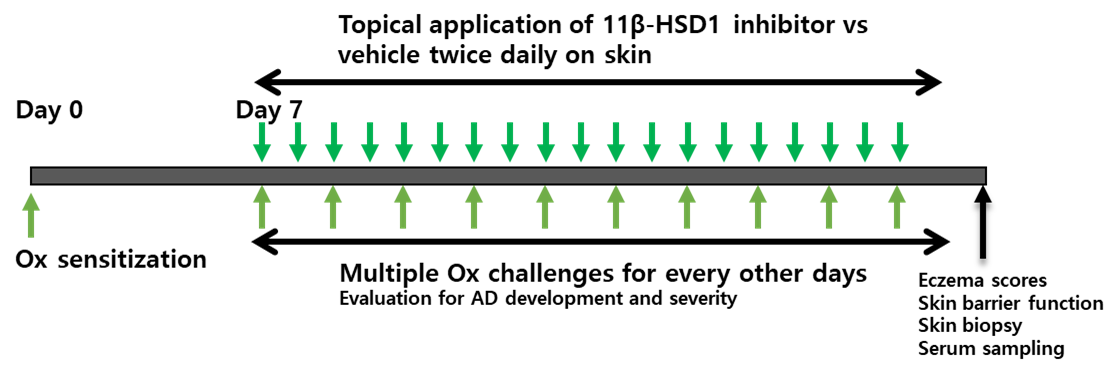


**Supplementary Figure 2. Topical Ox induction of AD-like dermatitis**

Mice were sensitized with one application of 1% Ox then topically challenged from one week later with 0.1% Ox every other day for 20 days. Selective inhibitor of 11β-HSD1 (385581)or DMSO vehicle was concurrently to both sides of the AD-induced mice (n = 7 per group) twice daily. Acetone was applied to control mice (n=5). Eczema was scored at each challenge from 5 to 10. TEWL and SC hydration, and well as SC pH were determined before sacrifice.

**Supplementary Figure 3. Circadian rhythm of serum corticosterone in *HSD11B1* KO mice**

Serum corticosterone levels in one *HSD11B1 KO* mouse per time point were measured using ELISA at 09:00, 15:00, 18:30, and 21:00 h. Data are presented as means of triplicate reads of each sample.

**Supplementary References**

1. Hau, C. S., Kanda, N. & Watanabe, S. Suppressive effects of antimycotics on thymic stromal lymphopoietin production in human keratinocytes. *J Dermatol Sci* **71,** 174-183, <https://doi.org/10.1016/j.jdermsci.2013.04.023> (2013).
